# Supplementary material for: The Scania Accelerated Intermittent Theta-burst Implementation Study (SATIS)–Lessons from an accelerated treatment protocol
Source: PLoS One. 2025 Jan 2;20(1):e0316339. doi: 10.1371/journal.pone.0316339 (PMC11694994; doi:10.1371/journal.pone.0316339)
Supplement: S1 Table — (PDF) [file pone.0316339.s001.pdf]

	Antidepressants	Moodstabilizers	Anxiolytics	Antipsychotics	Hypnotics	Stimulants
**1**	Buproprion	Lithium sulfate		Chlorprothixene	Zopiclone	
**2**	Amitriptyline	Lithium sulfate		Quetiapine	Zopiclone, Propiomazine	
**3**	Sertraline	Lithium sulfate				
**4**				Aripiprazole		
**5**	Citalopram, Mirtazepine				Zopiclone	
**6**	Agomelatine					
**7**	Duloxetine, Bupropion				Zopiclone, Propiomazine	
**8**	Nortriptyline					
**9**	Sertraline, Agomelatine	Lithium sulfate, Lamotrigine		Quetiapine	Zopiclone, Propiomazine	
**10**	Nortriptyline		Alimemazine		Zopiclone	Metylphenidate
**11**	Bupropion				Melatonin, Propiomazine	
**12**	Bupropion,Sert-raline, Mirtazapine					
**13**	Duloxetine, Agomelatine		Oxazepam		Zopiclone	Lisdexamphetamine
**14**	Venlafaxine	Lamotrigine	Diazepam			
**15**	Citalopram, Bupropion					
**16**	Venlafaxine, Mirtazapine		Alimemazine, Promethazine		Melatonin, Propiomazine	
**17**	Venlafaxine					
**18**				Quetiapine		
**19**	Escitalopram, Mirtazapine		Oxazepam		Zopiclone	
**20**	Fluoxetine				Melatonin	
