# Supplementary material for: The Scania Accelerated Intermittent Theta-burst Implementation Study (SATIS)–Lessons from an accelerated treatment protocol
Source: PLoS One. 2025 Jan 2;20(1):e0316339. doi: 10.1371/journal.pone.0316339 (PMC11694994; doi:10.1371/journal.pone.0316339)
Supplement: S2 Table — AEs reported by only one patient: Toothache, right arm paresthesia, transient hypersomnia, light-headedness, jaw discomfort during mastication, panic attack, supraorbital pain, tremor of the hands, slight spatial disorientation, bilateral shoulder myalgia, warm sensation under the coil, vertigo during treatment, buzzing in the head, feeling dazed, anticipatory anxiety, tension in the head, renal colic. (PDF) [file pone.0316339.s002.pdf]

**Reported adverse events**	**Number of patients**
Fatigue	7
Discomfort at treatment site	5
Nausea	4
Subjective postural instability	3
Subjective muscular weakness	2
Migraine	2
Blurred vision	2
Retro-orbital pressure	2
Supra-orbital pressure	2
Heavy-headedness	2
Soreness	2
