# Supplementary figures and images for: The Scania Accelerated Intermittent Theta-burst Implementation Study (SATIS)–Lessons from an accelerated treatment protocol
Source: PLoS One. 2025 Jan 2;20(1):e0316339. doi: 10.1371/journal.pone.0316339 (PMC11694994; doi:10.1371/journal.pone.0316339)

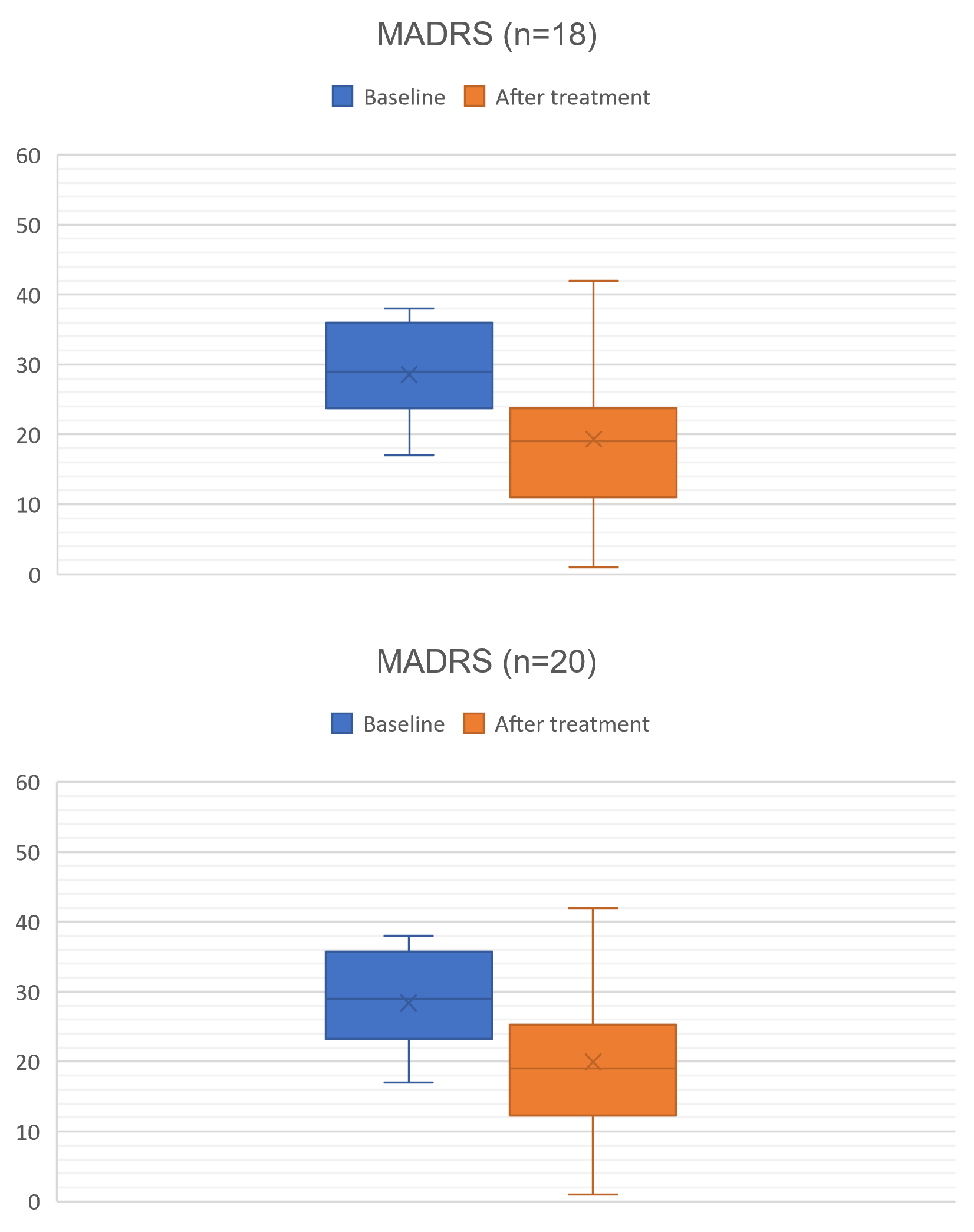

Supplement: S1 Fig — Whiskers min—max, box median, 1st and 3rd quartile. X marks the mean. Bottom: Depression score before and after treatment in the ITT group (n = 20). Whiskers min—max, box median, 1st and 3rd quartile. X marks the mean. (TIF) [file pone.0316339.s003.tif]

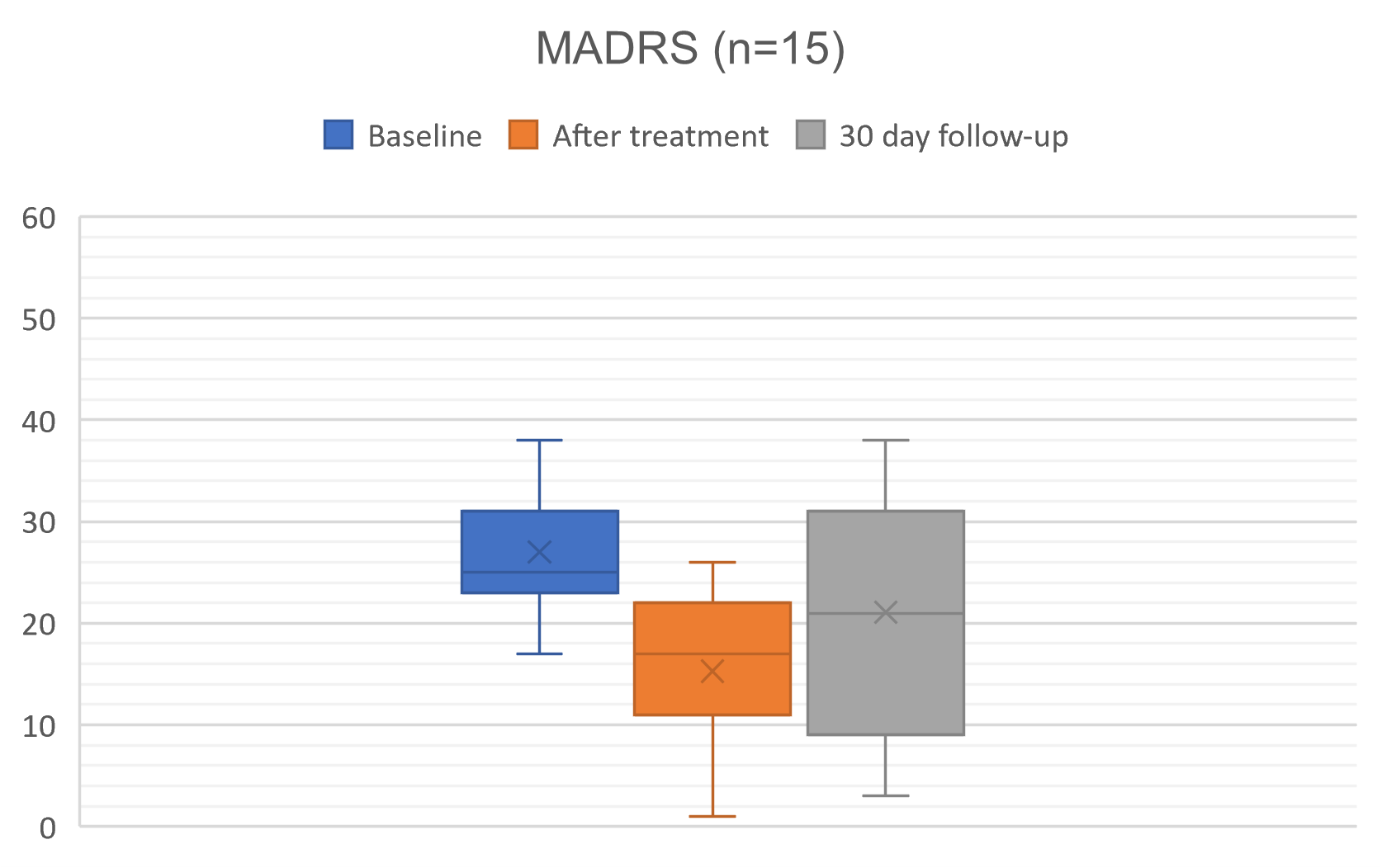

Supplement: S2 Fig — Whiskers min—max, box median, 1st and 3rd quartile. X marks the mean. (TIF) [file pone.0316339.s004.tif]

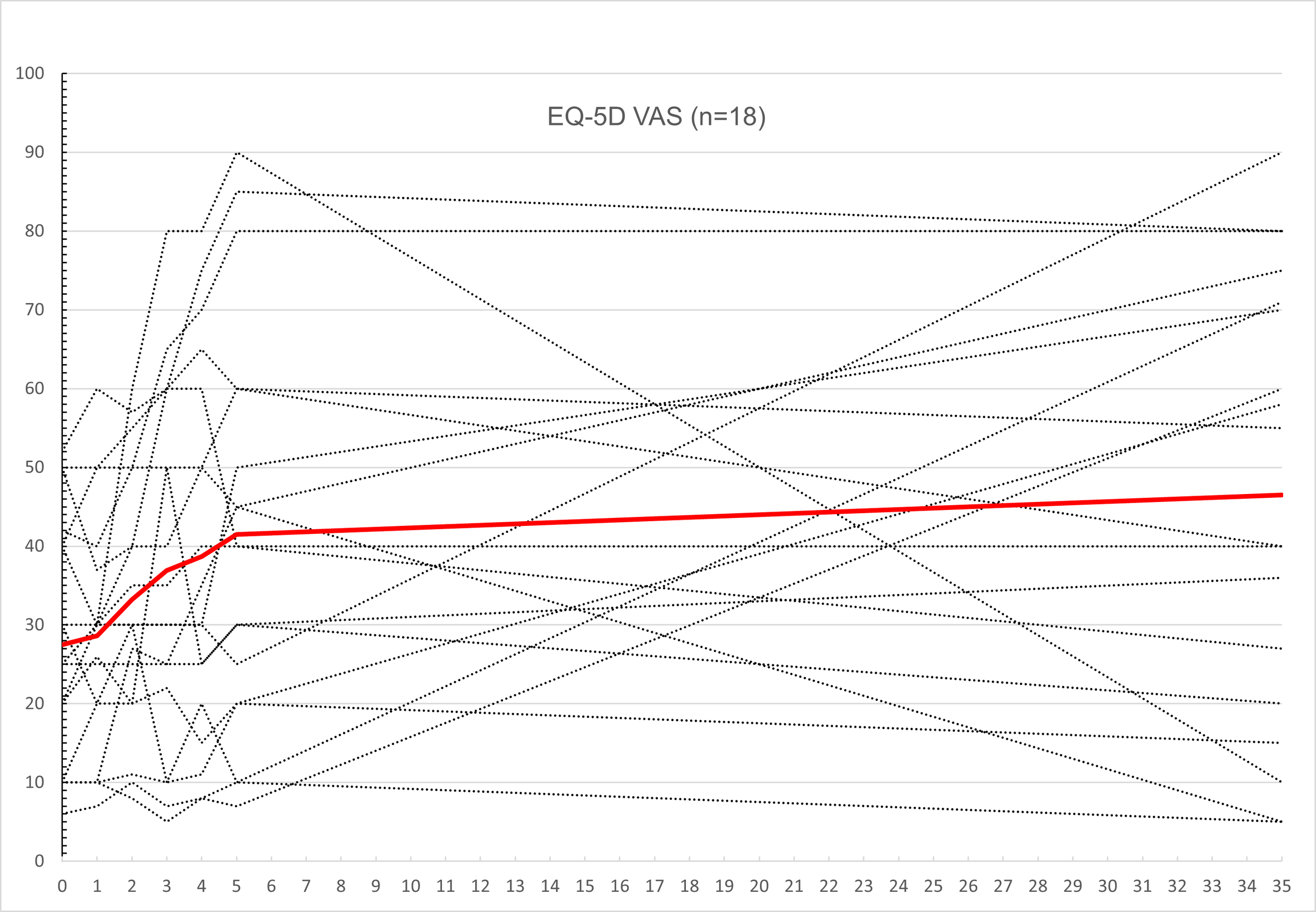

Supplement: S3 Fig — (TIF) [file pone.0316339.s005.tif]

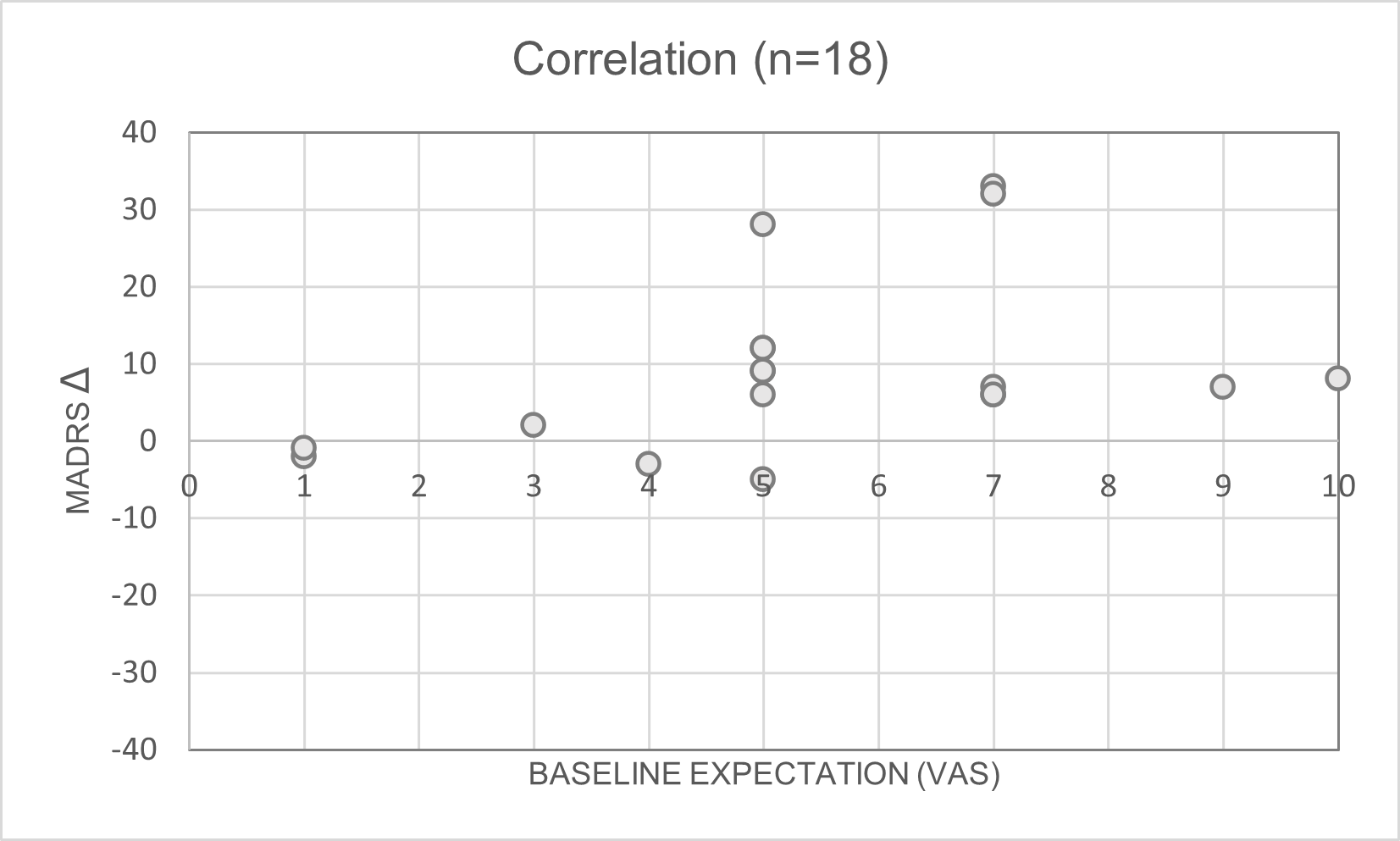

Supplement: S4 Fig — The three outliers represent patients with a very robust treatment response—a reduction in Montgomery-Åsberg depression rating scale (MADRS)-score of around 30 points. (TIF) [file pone.0316339.s006.tif]
