# Supplementary material for: The Scania Accelerated Intermittent Theta-burst Implementation Study (SATIS)–Lessons from an accelerated treatment protocol
Source: PLoS One. 2025 Jan 2;20(1):e0316339. doi: 10.1371/journal.pone.0316339 (PMC11694994; doi:10.1371/journal.pone.0316339)
Supplement: Attachment — Submitted filename: PLOS one_aTMS.docx [file pone.0316339.s008.docx]

1. In common TMS trials, participants with a high risk of suicide are often excluded, as well as those with psychotic features. Due to concerns about heterogeneity, cases with bipolar depression may also be excluded, or the trials may specifically focus on including only those with bipolar depression. Additionally, it is relatively rare to include cases with comorbid autism. Many trials also focus on treatment-resistant depression (TRD), most commonly defined as inadequate response to two antidepressants. Overall, these trials are usually designed this way to enhance the homogeneity of the participants. In this study, despite the small sample size and the high heterogeneity of the population, it is acceptable that the TMS outcomes are closer to those observed in national registry data rather than in a well-designed SAINT protocol study. This is actually more reflective of real-world clinical practice with TMS. However, the author should mention the limitations regarding the participants.

2. In addition, please describe the participants' medication usage status.

3. I would like to know the rationale behind designing the study with 5-7-7-7-7 sessions. Why not have 7 sessions each day for 5 days or 5 sessions each day? I suspect the author aimed to reduce the total sessions while increasing the MT% to compare with the SAINT Protocol. However, this should be explained in the Introduction.

4. Line 360 mentions that SAINT, with its higher number of sessions and total pulses, might be related to better outcomes. There is a considerable amount of research on TMS total pulses/total sessions and treatment effects (though not necessarily in the context of accelerated protocols). The author should delve deeper into this aspect—does more frequent stimulation necessarily lead to better effectiveness?

(PMID: 38183740, PMID: 38723735)

5. In the same vein, Line 373, The author mentions that the SAINT protocol uses a lower MT%, which is associated with better outcomes (??). This aspect also requires a more thorough discussion.

6. It is generally believed that the placebo effect can be quite significant in rTMS research (PMID: 29111404). In this study, many cases showed an immediate response within 2-3 days. Has the possibility of the placebo effect been considered?
